# 769. Risk Factors of Central Line-associated Bloodstream Infection in an Academic Medical Center

**DOI:** 10.1093/ofid/ofab466.966

**Published:** 2021-12-04

**Authors:** Zane Conrad, Minji Kang, Elizabeth Thomas, Doramarie Arocha, Julie B Trivedi

**Affiliations:** 1 UT Southwestern, Dallas, Texas; 2 UT Southwestern Medical Center - University Hospitals, Dallas, TX; 3 UT Southwestern Medical Center, Dallas, TX

## Abstract

**Background:**

Central line-associated bloodstream infections (CLABSI) are one of the leading healthcare-acquired infections (HAI) with significant morbidity and mortality. We aimed to identify risk factors of CLABSI at an academic medical center to determine high-risk populations and target interventions.

**Methods:**

This is an observational retrospective cohort study at William P. Clements Jr. University Hospital from January 1, 2017 to December 31, 2020. Retrospective chart review was conducted to identify demographics and co-morbidities of hospitalized patients diagnosed with CLABSI as defined by National Healthcare Safety Network (NHSN). Infections due to mucosal barrier injuries were excluded. Means were compared using independent-samples T-test and proportions were compared using chi-square.

**Results:**

Ninety-three CLABSI events were identified with an increase in the standardized infection ratio from 0.38 in 2017 to 0.74 in 2020 (Figure 1). Bacterial organisms were identified in 71 (76%) cases while fungal organisms were identified in 22 (24%) (Table 2). There was no significant difference in the timing of CLABSI after line insertion (p=0.09) or organism identified (p=0.61) in PICC lines (n=33, 34%) vs all other central lines (n=60, 67%). When comparing immunocompromised patients with CLABSI (n=47, 51%) vs non-immunocompromised (n=46, 50%), there was a significant difference in the indication for line (chemotherapy), but no difference was seen in the number of line days prior to event (p=0.57), line type (p=0.17), or organism identified (p=0.94). Of all CLABSI, 46% (n=43) were in the intensive care unit (ICU) with significantly more Candida species (p=0.018) identified compared to non-ICU patients with CLABSI (n= 50, 54%).

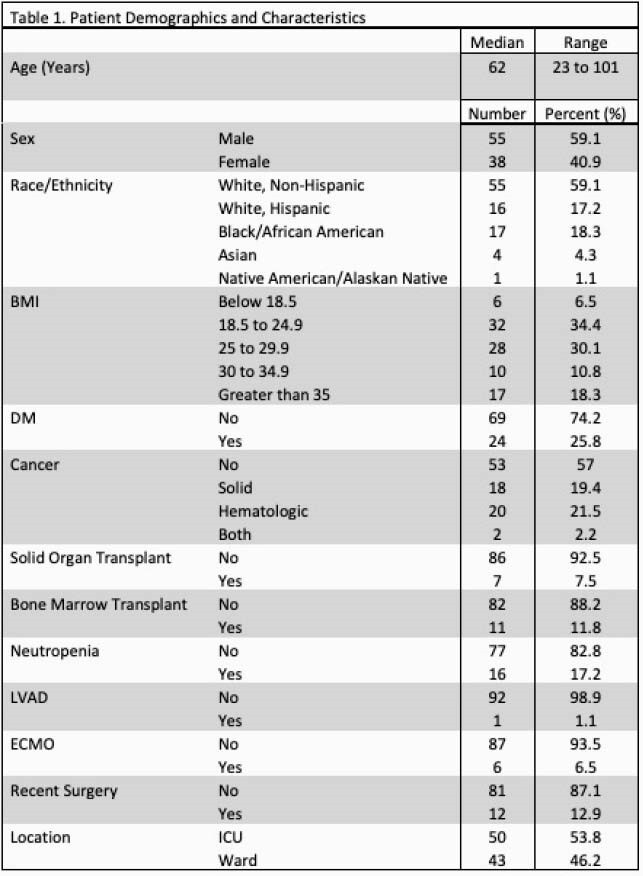

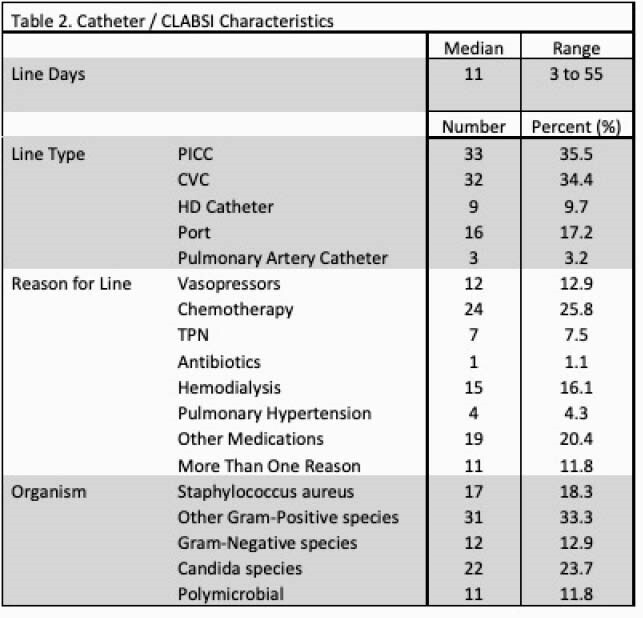

Figure 1. CLABSI Rate and SIR from 2017 to 2020 by Quarter

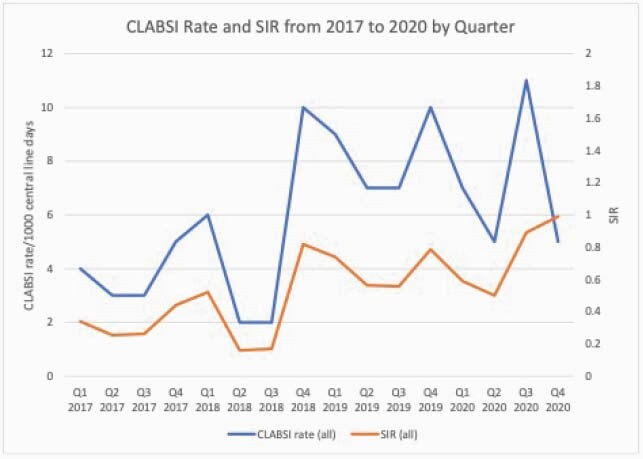

**Conclusion:**

Candida species were more likely to be found in ICU patients with CLABSI as compared to non-ICU counterparts with further investigation in the ICU population revealing lack of flushing after administration of total parenteral nutrition. Otherwise, this observational cohort of CLABSI events did not identify any difference in immunosuppression status or line type. Given this information, infection prevention efforts will continue to be directed towards proper central line maintenance and removal when no longer indicated.

**Disclosures:**

**All Authors**: No reported disclosures

